# Efficacy and safety of oxygen-sparing nasal reservoir cannula for treatment of pediatric hypoxemic pneumonia in Uganda: a pilot randomized clinical trial

**DOI:** 10.1186/s12890-020-01267-8

**Published:** 2020-08-31

**Authors:** Jerry Mulondo, Stella Maleni, Hellen Aanyu-Tukamuhebwa, Ezekiel Mupere, Alfred Onubia Andama, Chin Hei Ng, Stephen Burkot, Ella M. E. Forgie, Qaasim Mian, Christine M. Bachman, Gerard Rummery, Daniel Lieberman, David Bell, Michael T. Hawkes, Akos Somoskovi

**Affiliations:** 1 Infectious Diseases Research Collaboration, Kampala, Uganda; 2 Department of Pediatrics and Child Health, Mulago National Referral Hospital and Makerere University, Kampala, Uganda; 3 Department of Pediatrics, Makerere University College of Health Sciences, Kampala, Uganda; 4 Department of Medicine, Makerere University College of Health Sciences, Kampala, Uganda; 5 Intellectual Ventures, Global Good Fund, Bellevue, WA USA; 6 Department of Pediatrics, University of Alberta, 3-588D Edmonton Clinic Health Academy, 11405 87 Ave NW, Edmonton, Alberta T6G 1C9 Canada; 7 ResMed Ltd., Bella Vista, Australia; 8 Present address: Issaquah, USA; 9 Department of Medical Microbiology and Immunology, University of Alberta, Edmonton, Canada; 10 School of Public Health, University of Alberta, Edmonton, Canada; 11 Stollery Science Lab, Edmonton, Canada; 12 Women and Children’s Health Research Institute, Edmonton, Canada

**Keywords:** Oxygen, Pneumonia, Africa, Child, Nasal Canula

## Abstract

**Background:**

Oxygen is an essential therapy for hypoxemia but is scarce in low-income settings. Oxygen conserving devices optimize delivery, but to date have been designed for adults in high-income settings. Here we present the development and clinical pilot study of an oxygen-sparing nasal reservoir cannula (OSNRC) for pediatric use in low-income settings.

**Methods:**

(1) Pre-clinical development of a novel OSNRC using a simulated respiratory circuit with metabolic simulator and anatomically accurate face-airway models. Simulated breathing waveforms were designed based on airway resistance, lung compliance, respiratory rate, and tidal volume of spontaneous breathing for three disease conditions. (2) Pilot, randomized, controlled, non-blinded, cross-over study of the OSNRC vs standard nasal cannula (SNC) among children hospitalized with hypoxemic pneumonia in Uganda. Eight children were randomized to OSNRC followed by SNC, and eight were randomized to SNC followed by OSNRC.

**Results:**

The laboratory simulation showed that the OSNRC provided the same or higher fraction of inspired oxygen at approximately 2.5-times lower flow rate compared to SNC. The flow savings ratio exhibited a linear relationship with the OSNRC volume to tidal volume ratio with a slope that varied with breathing waveforms. The range of performance from different breathing waveforms defined a performance envelope of the OSNRC. Two mask sizes (30 mL and 50 mL) provided sufficient coverage for patients between the 3rd and 97th percentile in our targeted age range. In the clinical pilot study, the rise in capillary blood pCO_2_ was similar in the OSNRC and SNC groups, suggesting that the OSNRC was not associated with CO_2_ retention. There were no significant differences between OSNRC and SNC with respect to clinical adverse events, lactate levels, pH, and SpO_2_. The OSNRC group had a higher mean SpO_2_ than the SNC group (adjusted mean difference, 1.4, 95% confidence interval 1.1 to 1.8), showing oxygen delivery enhancement.

**Conclusion:**

The OSNRC enhances oxygen delivery without causing CO_2_ retention and appears to be well-tolerated by pediatric patients. If safety, efficacy and tolerability are confirmed in larger trials, this device has the potential to optimize oxygen delivery in children in low-resource settings, reducing the global burden of pediatric pneumonia.

**Trial registration:**

The trial was retrospectively registered (International Standard Registered Clinical/Social Study Number (ISRCTN): 15216845; Date of registration: 15 July 2020).

## Background

Pneumonia is the leading cause of death among children under 5 years old globally [1, 2], accounting for 15% of all childhood deaths [3]. Hypoxemia is a potentially fatal complication of pneumonia, and the risk of death increases with increasing severity of hypoxemia [2, 4–6].

Oxygen is an essential supportive treatment of hypoxemia, and reduces the mortality associated with severe pneumonia [7, 8]. However, deficiencies in the delivery and sustainability of oxygen to hypoxemic infants and children vary significantly in resource-limited settings [9]. Compressed oxygen cylinders are widely used but they may be expensive and difficult to transport, requiring regular replenishment and a functional supply chain [10]. Oxygen concentrators generate oxygen on site and may be less expensive; however, their use requires an uninterrupted power supply [8]. Central piped oxygen requires costly infrastructure that may be impractical for most hospitals in resource-limited countries. Medical grade oxygen is thus scarce in low-income settings.

Several methods are available for attaching oxygen to the patient [8]. Nasal prongs are optimal in terms of safety and efficacy, and are widely used in hospitals globally. Nasal or nasopharyngeal catheters are alternatives recommended by the World Health Organization (WHO) [8]. Face­masks, head boxes, incubators and tents may also be used, but are associated with oxygen wastage and may not be appropriate where oxygen is in short supply.

Oxygen conserving devices (OCDs) function by changing the interface (e.g., reservoirs and trans-tracheal catheters) or changing the oxygen delivery system (e.g., demand oxygen delivery systems, DODS). These devices achieve a flow savings ratio (FSR), defined as the ratio of oxygen flow rate using a standard nasal cannula (SNC) to the oxygen flow rate with the device which produces the same clinical effect. Several previous studies have examined different oxygen-conserving techniques, primarily developed for adult patients with chronic hypoxemia due to chronic obstructive pulmonary disease (COPD), to reduce costs of long-term oxygen therapy. The principles underlying oxygen-sparing include: bypassing the dead space of the upper airway (e.g., surgically implanted transtracheal catheters) [11–13]; interrupting the flow during exhalation (e.g., DODS); and storing exhaled oxygen in a reservoir to make it available at the next inhalation (e.g., Pendant Conserving Nasal Cannula, PNC) [14, 15]. Examples of DODS include AccuO2 and CR-50, which can achieve FSRs of 9.9 ± 7.3 and 2.6 ± 1.0, respectively [16]. Reservoir masks, a common OCD, are used in combination with a SNC, and they are available for clinical use. Storing oxygen in the reservoir space during exhalation allows the patient to inspire a higher concentration of oxygen without increasing flow from the oxygen tank or concentrator at the next inhalation. This enables a reduction in the oxygen flow while maintaining the same peripheral oxygen saturation (SpO_2_), reducing the overall volume needed and cost of oxygen therapy [17]. However, re-breathing exhaled carbon dioxide (CO_2_) is a potential limitation of this strategy that could lead to hypercarbia and acidosis. Oxygen conserving reservoirs could be a simple, yet important tool for improving the efficiency of oxygen delivery in resource limited settings [18]. The PNC is typically used in adults but not pediatric patients because of the relatively large dead space for the smaller tidal volume in children. However, this limitation may be overcome by placing the reservoir directly at the nose which reduces the volume of dead space in the excess tubing.

The objective of this study was to design and optimize an oxygen-sparing nasal reservoir cannula (OSNRC) and collect pilot data on its safety and efficacy among Ugandan children hospitalized with hypoxemic pneumonia. Our aim was to reserve highly saturated oxygen during exhalation to be re-inhaled, while minimizing re-intake of exhaled CO_2_.

## Methods

### Experimental apparatus

We hypothesized that the RC would reduce the flow rate of oxygen required to deliver an equal or higher FiO_2_ per oxygen delivered (L/min) compared with SNC alone. We speculated that the primary disadvantage of the RC was associated with increased CO_2_ levels in exhaled air that could potentially lead to elevated toxicity levels. We tested this hypothesis using an experimental respiratory circuit.

**Fig. 1:**
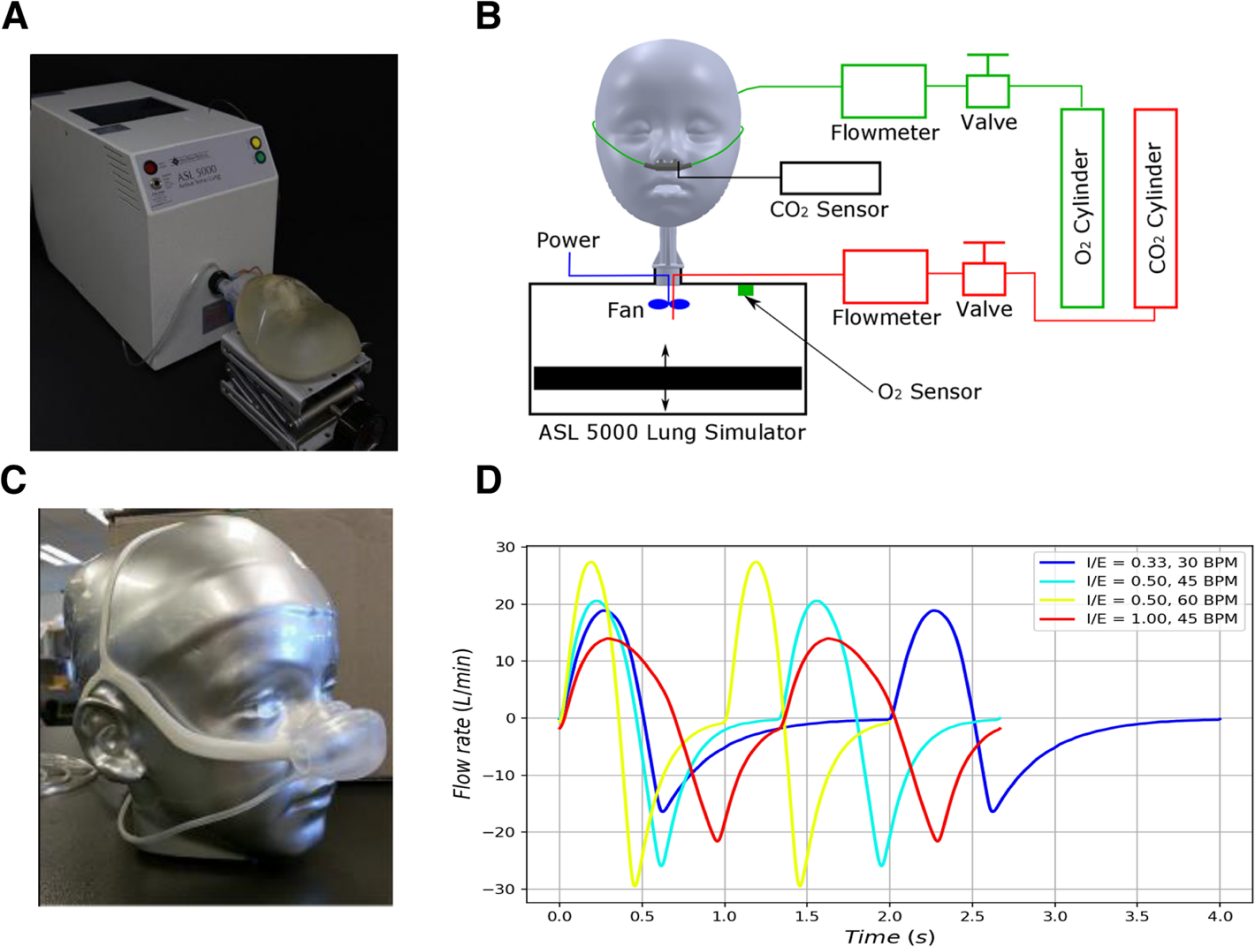
Experimental apparatus for the pre-clinical design and testing of oxygen-sparing nasal reservoir cannula (OSNRC). **a.** Image of experimental breathing circuit, consisting of OSNRC, SNC, model face and upper airway, coupled with a breathing simulator. **b.** Schematic of the experimental apparatus. **c.** Image of OSNRC placed on 3D printed anatomically accurate face model. The OSNRC fits over a standard nasal cannula (SNC). **d.** Breathing waveforms (normal and diseased conditions) used to represent a 12 kg patient. Inspiratory to expiratory (I/E) ratios ranged from 0.33–1 and respiratory rate ranged from 30 to 60 breaths per minute (BPM)

An image and a schematic of the experimental apparatus are shown in Fig. 1. A series of anatomically accurate face-airway models were created by digitally combining three-dimensional (3D) images of faces [19] and CT scan of airways from the nostrils to the trachea [20]. 3D printed versions of the models, representing newborns to 5-year-olds, coupled with the ASL 5000 breathing simulator (IngMar Medical, Pittsburgh, PA) formed the complete breathing circuit (Fig. 1b).

Oxygen was supplied through a SNC or an OSNRC from standard medical grade oxygen cylinders. Oxygen flow rate was recorded using TSI 4040 flowmeter (TSI, Shoreview, MN). A built-in oxygen sensor recorded oxygen concentration inside the breathing simulator. Metabolic production of CO_2_ was simulated in the system by injecting CO_2_ through a port of the breathing simulator. End-tidal CO_2_ (ETCO_2_) was measured at the nostril using the Oxigraf O2Cap (Oxigraf, Inc., Sunnyvale, CA). The CO_2_ production rate for each patient age was set by titrating the CO_2_ flow rate until the ETCO_2_ reached 5% under conditions of spontaneous breathing. The CO_2_ flow rate was quantified using an Alicat M-10SLPM-D/5 M mass flowmeter (Alicat Scientific, Tucson, AZ). The empirically determined CO_2_ production rate was applied to all experiments of the same patient age. All measurements were taken when the mean quantity within each breathing cycle reached steady state.

### Breathing waveforms

The simulated breathing waveforms were designed based on published values for airway resistance [21], lung compliance [21], respiratory rate [22], and tidal volume [23, 24] of spontaneous breathing (called normal). Breathing waveforms of three different disease conditions for each age were created using modified parameters (lung compliance, respiratory rate and inhalation-to-exhalation ratio). Examples of the breathing waveforms used in this study are shown in Fig. 1d.

### OSNRC volume sizing

The design of the OSNRC required us to balance several factors and determine appropriate sizes for the intended users. Larger mask volumes tend to provide larger FSRs at the risk of increasing CO_2_ retention and/or patient tolerability. We defined a minimum FSR of 1.8 and a maximum ETCO_2_ 9% under no flow conditions.

The FSR was defined as:

$$ \frac{Q_{SNC}}{Q_{OSNRC}}=\frac{Reference\ flow\ rate\ with\  SNC}{Equivalent\ flow\ rate\ with\ OSNRC} $$

where Q_SNC_ is the reference flow rate with SNC and Q_OSNRC_ is the flow rate with OSNRC to achieve the same fraction of inspired oxygen (FiO_2_) as using the SNC with the reference flow rate.

### Tolerability

As an initial assessment of the tolerability of the OSNRC for pediatric patients, a fit test was conducted on children without acute respiratory disease during their follow-up visits at the Chest Clinic of the Department of Pediatrics of Mulago Hospital in Kampala. Patients were examined with the prototype for up to 5 min. Information regarding placement, size and comfort was collected using a standardized questionnaire, provided in the Supplementary materials.

### Pilot clinical study

We conducted a pilot study of the OSNRC children hospitalized with hypoxemic pneumonia at a large resource-limited hospital in Uganda, Mulago National Referral Hospital. Details of the study are given in Supplementary materials. In brief, infants and children under the age of 5 years hospitalized with hypoxemia (SpO_2_ ≥ 85 and < 94%) were provided oxygen using the OSNRC for a period of 1 hour. As a control condition, each patient also received oxygen via SNC for another period of 1 hour. The order of OSNRC and SNC for each child was randomly assigned. Group A received oxygen using the OSNRC for Period 1, followed by SNC for Period 2. Group B received oxygen using the SNC for Period 1, followed by the OSNRC for Period 2. The flow rate of oxygen began at 1.5 to 2 L/min and was systematically titrated downward, as allowed, to maintain SpO_2_ > 94%. Vital signs were recorded every 15 min. At the end of each hour, capillary blood gas (pH, pCO_2_, pO_2_, base excess, HCO_3_ and lactate) was measured. Any adverse events were also noted. The primary focus of the trial was clinical safety of the OSNRC.

As the primary safety outcome, we examined CO_2_ retention, as measured by the change in pCO_2_ after 1 h on OSNRC versus SNC alone (Period 1). Secondary safety outcomes included: clinical adverse events, capillary blood gas pCO_2_ above normal range (> 45 mmHg), lactate above normal range (> 3 mmol/L), acidosis (pH < 7.35), and refractory hypoxemia (SpO_2_ < 90%) despite supplemental O_2_ therapy at any time on OSNRC. In addition, we compared the temporal trends in pCO_2_, pH, and lactate to the control group receiving O_2_ by SNC. Secondary efficacy outcomes included oxygen utilization and SpO_2_ at several O_2_ flow rates, compared between OSNRC and SNC. These outcomes were specified a priori in the trial protocol. Capillary blood gas was measured using an iSTAT-1 handheld analyzer with CG4+ cartridges (Abbott Point of Care Inc., Princeton, NJ). Pulse oximetry employed the Rad-5® oximeter (Masimo Corp., Irvine, CA).

## Results

### Pre-clinical evidence of oxygen sparing

**Fig. 2:**
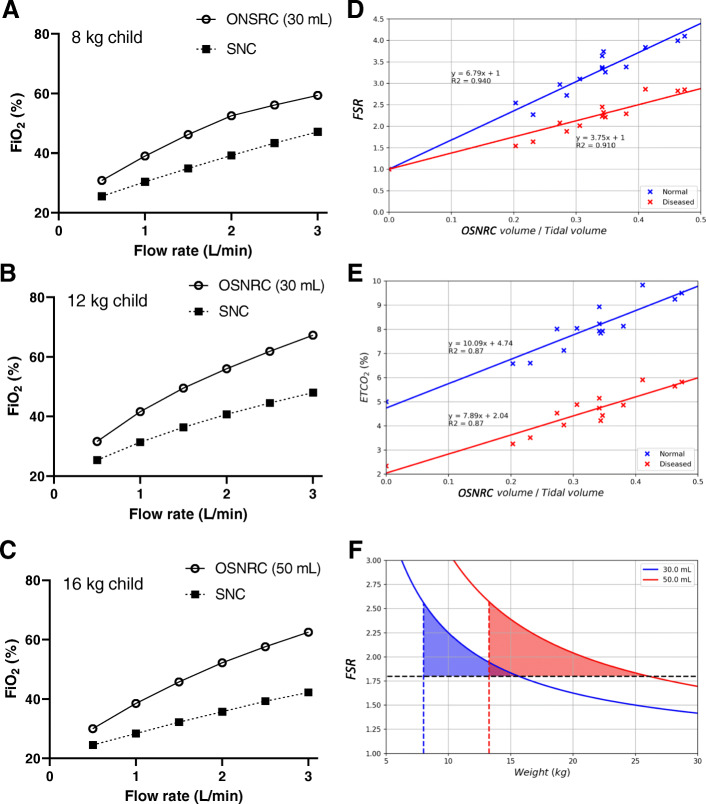
Design of oxygen-sparing nasal reservoir cannula (OSNRC) based on oxygen sparing and CO_2_ retention. **a-c**. The fraction of inspired oxygen (FiO_2_) was higher at a given flow rate for the OSNRC (circle, solid line) compared to the SNC (square, dashed). The breathing simulator used a flow waveform resembling a patient with pneumonia. The tidal volume approximated that of an 8 kg (**a**), 12 kg (**b**) and 16 kg child (**c**), respectively. Two OSNRC sizes were used: 30 mL (**a** and **b**) and 50 mL (**c**). Data shown represent results from a single simulation at each condition. The flow savings ratio (FSR) was calculated to be 1.8 to 2.6. **d**. FSR as a function of OSNRC volume to tidal volume ratio. **e**. End-tidal CO_2_ (ETCO_2_), measured at the outlet of the simulated respiratory circuit, as a function of OSNRC volume to tidal volume ratio, using constant age-appropriate CO_2_ production in the circuit. **f**. OSNRC sizes (30 mL and 50 mL) were designed for patients from 8 kg to 26 kg. Sizes were bounded by minimum FSR of 1.8 (horizontal dashed line), maximum ETCO_2_ of 9% (vertical dashed lines), and maximum FSR observed (solid lines). Colored regions represent the range of operating states of the OSNRC

Using our experimental respiratory circuit (Fig. 1), the OSNRC provided higher FiO_2_ at different flow rates, simulated patient sizes, and mask sizes (Fig. 2a-c). The same FiO_2_ could be obtained with a lower flow rate when using the OSNRC, with a FSR of 1.8 to 2.6 under these conditions (Fig. 2a-c). The FSR (Q_SNC_/Q_OSNRC_) was measured for a range of OSNRC volumes between 14 and 60 mL and for different breathing waveforms. The FSR varied between 1.5 and 4 and exhibited a linear relationship with the OSNRC volume to tidal volume ratio (Fig. 2d). The slope of this linear regression depended on the breathing waveforms (Fig. 2d).

The range of performance from different breathing waveforms defined a performance envelope of the OSNRC. The risk of elevated ETCO_2_ was evaluated with no oxygen flow, representing the worst-case condition. Similar to the FSR, ETCO_2_ also scaled linearly with the OSNRC volume to tidal volume ratio (Fig. 2e).

Both FSR and ETCO_2_ data were then used to determine the OSNRC inner volume for target patient demographics. We found that two mask sizes 30 mL and 50 mL would be sufficient to cover patients weighing between 8 to 26 kg (Fig. 2f), which corresponds to patients between the ages of 18 and 66 months, using the 3rd and 97th percentile for weight [24]. The smaller mask size could be used on participants weighing 8–13 kg, while the larger mask size could be used on participants weighing 14–26 kg.

### Tolerability

A fit test was performed on 6 patients (3 per OSNRC size) without respiratory disease as an initial assessment of the tolerability of the device (Table 1). The median (IQR) age in months was 37 (33–43) and 5 participants (83%) were male. The reservoir was appropriate in size for all 6 patients and the head band offered additional support for the device. The OSNRC was well-tolerated by 5 (83%) of the fit test participants. The patient who did not tolerate the OSNRC also did not tolerate the SNC.

**Table 1:** Tolerability and fit testing of OSNRC

	Cohort (***n*** = 6)
**Demographics**	
Male sex, n (%)	5 (83)
Age (months), median (IQR)	36 (34–41)
Weight, median (IQR)	14 (12–16)
Mask size	
30 mL	3 (50)
50 mL	3 (50)
**Tolerability, n (%)**	
Mask fits appropriately	5 (83)
Patient can tolerate mask	5 (83)
Mask placement easy to perform	6 (100)
Patient can tolerate face band	5 (83)
Face band placement easy to perform	6 (100)
Mask size appropriate	6 (100)

### Clinical safety of nasal reservoir cannula

**Fig. 3:**
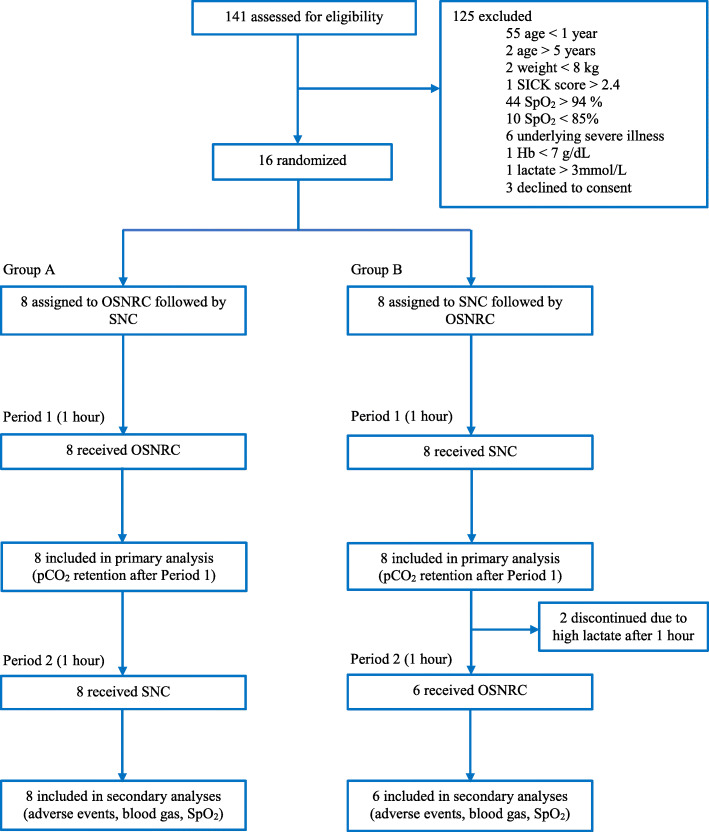
Trial profile. Pilot study of oxygen sparing nasal reservoir cannula (OSNRC) versus standard nasal cannula (SNC) among Ugandan children hospitalized with hypoxemia (*n* = 16). The flowchart shows the two trial Periods 1 and 2, and the treatment received by patients in each Group A (OSNRC first, then SNC) and Group B (SNC first, then OSNRC) during each period. Two patients in Group B discontinued the trial after Period 1 (SNC) due to hyperlactatemia

To demonstrate safety, we next piloted the OSNRC in a small group of children with hypoxemia in a resource-limited setting. Sixteen participants were recruited between November 20, 2018 and May 24, 2019. The pilot study flow diagram is shown in Fig. 3. Baseline characteristics are shown in Table 2.

**Table 2:** Baseline characteristics of participants

	Entire Cohort (***n*** = 16)	Group A^**a**^ (***n*** = 8)	Group B^**b**^ (***n*** = 8)
**Demographics**			
Male sex (n, %)	8 (50)	5 (63)	3 (38)
Age (months), median (IQR)	23 (17–29)	20 (15–28)	26 (20–32)
**Past medical history**			
Pneumonia	6 (38)	2 (25)	4 (50)
Asthma	1 (6)	1 (13)	0
HIV	0	0	0
Malaria	3 (17)	0	3 (38)
**Clinical examination**			
Baseline SpO_2_	90 (88–93)	89 (88–92)	90 (87–93)
85–89%	8 (50)	4 (50)	4 (50)
90–94%	8 (50)	4 (50)	4 (50)
Weight (kg), median (IQR)	11.4 (9.0–13.0)	11.5 (9.0–13.1)	10.9 (9.5–12.6)
Temperature (°C), median (IQR)	37.4 (37–37.9)	37.4 (37.1–37.5)	37.9 (37–38.1)
Blood pressure (mmHg)^3^			
Systolic, median (IQR)	95 (92–101)	96 (87–104)	95 (92–100)
Diastolic, median (IQR)	67 (62.5–78.5)	65 (60–72)	73 (65–80)
Heart Rate (bpm), median (IQR)	157 (138–168)	152 (142–160)	159 (135–179)
Respiratory rate (bpm), median (IQR)	63 (57.5–76)	66 (60–76)	63 (54–76)
Tachypnea	16 (100)	8 (100)	8 (100)
Delayed Capillary refill time	0	0	0
Absent or unequal breath sounds	0	0	0
Wheeze	1 (8)	0	1 (6)
Crackles	12 (75)	6 (75)	6 (75)
Stridor	0	0	0
Rapid or shallow breathing	16 (100)	8 (100)	8 (100)
Increased work of breathing	16 (100)	8 (100)	8 (100)
Chest wall asymmetry	0	0	0
Consciousness			
Alert	16 (100)	8 (100)	8 (100)
Response to Voice	0	0	0
Response to pain	0	0	0
Unresponsive	0	0	0
SICK scores	2.1 (0.9–2.2)	1.5 (0.9–2.1)	2.1 (1.4–2.3)
**Investigations, median (IQR)**			
Venous blood gas			
Lactate (mmol/L)	1.8 (1.54–1.9)	1.9 (1.8–2)	1.7 (1.4–2.0)
pH	7.4 (7.4–7.5)	7.4 (7.4–7.5)	7.5 (7.4–7.5)
pCO_2_ (mmHg)	27 (24–32)	27 (25–32)	28 (24–31)
paO_2_ (mmHg)	44 (43–47)	43 (42–46)	45 (43–49)
BE (mmol/L)	-5 (−8 to − 4)	−6 (− 9 to − 5)	−5 (− 6 to − 2)
HCO3 (mmol/L)	19 (17–20)	18 (16–20)	20 (18–21)
Blood glucose (mmol/L)	6 (5.2–6.9)	5.6 (5.1–6.5)	6.5 (5.5–7.0)
Hematologic parameters			
Hemoglobin (g/dL)	11.1 (10–11.8)	11 (10–12.2)	11.1 (10–11.5)
Hematocrit (%)	35 (31–36)	34 (31–36)	34 (31–36)
White blood cell count (x10^3^μL)	13 (8–18)	10 (8–21)	15 (7–18)
Platelet count (×10^3^ μL)	396 (271–552)	424 (320–552)	396 (260–540)

Data represent n (%) unless otherwise specified

*IQR* Interquartile Range

^a^Group A received OSNRC during Period 1, followed by SNC during Period 2

^b^Group B received SNC during Period 1, followed by OSNRC during Period 2

**Fig. 4:**
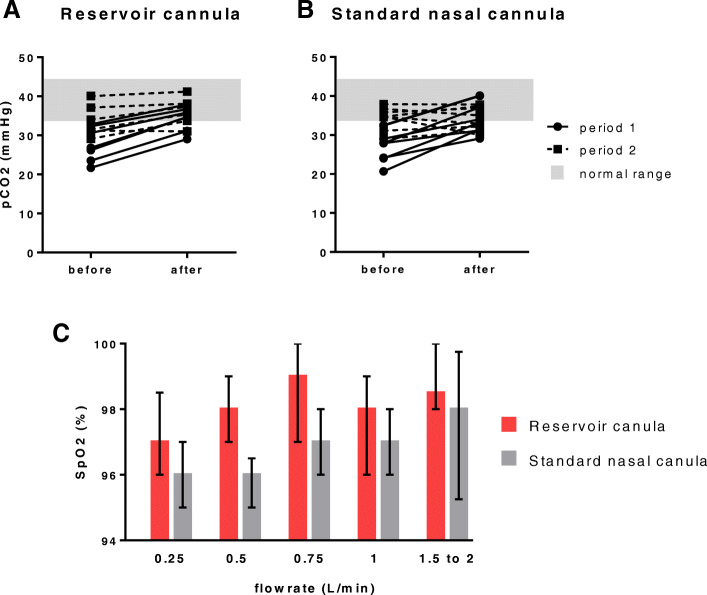
Clinical pilot data comparing oxygen-sparing nasal reservoir cannula (OSNRC) and standard nasal cannula (SNC) in hypoxemic Ugandan children. **a** and **b**. Normalization of hypocapnia with resolving tachypnea was observed in patients using both OSNRC and SNC, with no evidence of greater CO_2_ retention in the OSNRC group relative to the SNC group. **c** and **d**. Capillary blood gas pH changes were similar in OSNRC and SNC groups. **e**. Evidence of oxygen sparing by the OSNRC. Peripheral oxygen saturation (SpO_2_) increased with increasing oxygen flow rate in patients using both OSNRC and standard nasal cannula (SNC), but was comparatively higher at several flow rates with the OSNRC. In a linear mixed-effects model, the increase in SpO_2_ was 1.6% for each 1 L/min increase in flow rate and was 1.4% higher for OSNRC, relative to SNC (*p* < 0.0001)

With respect to our primary safety outcome, we did not observe evidence of CO_2_ retention during the first hour of treatment with the OSNRC (Fig. 4). The mean (SD) rise in capillary blood pCO_2_ with OSNRC was 7.2 (2.6) mmHg compared to 6.8 (1.6) with SNC alone (Fig. 4a and b, difference in means 0.43 mmHg, 95%CI − 2.8 to + 1.9). Secondary safety outcomes are shown in Table 3. Notably, no statistically significant differences in OSNRC versus SNC alone were detected for any of the safety endpoints. Two patients in Group B using the SNC were withdrawn according to protocol after Period 1 because their capillary blood gas lactate levels were higher than the pre-defined threshold for early discontinuation.

**Table 3:** Secondary safety outcomes

	Period^**a,b**^	OSNRC	SNC
**Clinical adverse event**	1	0	0
	2	0	0
**Severe adverse event**	1	0	0
	2	0	0
**pCO**_**2**_ **> 45 mmHg**	1	0	0
	2	0	0
**pH < 7.35**	1	2 (25)^c^	1 (12)
	2	1 (17)	2 (25)
**Lactate > 3 mmol/L**	1	0	2 (25)
	2	1 (17)^d^	0
**SpO**_**2**_ **< 90% despite O**_**2**_	1	0	0
	2	0	0

Data represent the number (percent) of patients who experienced an adverse event with OSNRC and SNC (control)

^a^During Period 1, patients were treated with OSNRC (Group A, *n* = 8) or SNC (Group B, *n* = 8) for 1 hour

^b^During Period 2, patients were treated with OSNRC (Group B, *n* = 6) or SNC (Group A, *n* = 8) for 1 hour

^c^*P* > 0.99 for difference between OSNRC and SNC treatments

^d^*P* = 0.43 for difference between OSNRC and SNC treatments

At admission, patients had evidence of compensated respiratory alkalosis, with low pCO_2_ (median 27 mmHg, IQR 24 to 30), and negative base excess (− 5 mmol/L, IQR − 8 to − 4, Table 2). A rise in the pCO_2_ toward normal levels was observed in patients on OSNRC, of median magnitude 7.5 mmHg (IQR 5.2 to 7.9) and 2.2 mmHg (IQR 1.1 to 3.7) during periods 1 and 2, respectively. This rise in pCO_2_ was similar in magnitude to patients on SNC (*p* > 0.1 for both period 1 and 2, Table 4). Of note, the rise in pCO_2_ was associated with a reduction in the respiratory rate reflecting the normalization of the minute ventilation, and a statistically (but not clinically) significant decrease in the pH, without significant change in the base excess (Table 4 and Fig. 4). Notably, the magnitude of the change in blood gas parameters was similar in patients with OSNRC and SNC (Table 4 and Figure S1, Supplemental materials). No patients with OSNRC or SNC developed hypercapnia or acidosis at any point in the study.

**Table 4:** Change in vital signs and capillary blood gas parameters with OSNRC or SNC over study periods 1 and 2 of crossover RCT

	Period^**a,b**^	OSNRC	SNC	***P***-value^**c**^
**SpO**_**2**_ **(%)**	1	− 1 (− 2 to − 0.75)	−1.5 (− 2.2 to − 1)	0.44
	2	− 1.5 (− 2.8 to − 1)	0 (−1 to 1.2)	0.1
**RR (min**^**−1**^**)**	1	−2 (− 6 to − 1)	0 (− 7 to 2)	0.7
	2	2 (2 to 4)	−3 (− 5 to 0.5)	0.1
**HR (min**^**−1**^**)**	1	−14 (− 16 to − 11)	1 (− 0.25 to 3.5)	0.0086
	2	3 (−5.2 to 6)	−4 (− 9.8 to 2.8)	0.48
**pCO**_**2**_ **(mm Hg)**	1	7.5 (5.2 to 7.9)	7.6 (5 to 9.2)	0.79
	2	2.2 (1.1 to 3.7)	0.5 (−1.7 to 1.3)	0.11
**pH**	1	− 0.063 (− 0.073 to − 0.041)	− 0.073 (− 0.11 to − 0.035)	0.44
	2	− 0.01 (− 0.018 to − 0.0065)	0.0025 (− 0.0092 to 0.0083)	0.34
**Lactate (mmol/L)**	1	− 0.48 (− 0.72 to − 0.32)	0.33 (− 0.4 to 0.98)	0.028
	2	−0.13 (− 0.15 to 0.13)	0.3 (0.1 to 0.43)	0.18
**Base excess (mmol/L)**	1	0.5 (0 to 2.2)	0 (0 to 0.25)	0.34
	2	0.5 (0 to 1)	−1 (− 1.2 to 0.25)	0.099

Data represent the median (interquartile range)

^a^During Period 1, patients were treated with OSNRC (Group A, *n* = 8) or SNC (Group B, *n* = 8) for 1 hour

^b^During Period 2, patients were treated with OSNRC (Group B, *n* = 6) or SNC (Group A, *n* = 8) for 1 hour

^c^Represents *p*-value for difference between OSNRC and SNC treatments

We also examined SpO_2_ as a function of flow rate for evidence of oxygen sparing with the OSNRC, relative to SNC alone (Fig. 4c). In a linear mixed-effects model accounting for repeated measures on individual patients, the SpO_2_ increased by 1.6% (95%CI 1.2–2.0, *p* < 0.0001) for every 1 L/min increase in flow rate, and was 1.4% (95%CI 1.1 to 1.8, *p* < 0.0001) higher in the OSNRC group compared to SNC group (Table 5). On visual inspection, the increase in SpO_2_ was most prominent at lower flow-rates, with no apparent difference at flow rates > 1 L/min (Fig. 4). The OSNRC was associated with an increase in SpO_2_ equivalent to an incremental flow rate increase of 0.9 L/min, and an FSR of 1.5.

**Table 5:** Random-intercept linear mixed effects model of saturation as a function of oxygen flowrate and OSNRC (versus SNC)

	Coefficient (95% CI)	***P***-value
**Fixed effects**		
**Flow rate**	1.6 (1.2–2.0)	< 0.0001
**Cannula**		
**SNC**	1.0 (reference)	
**OSNRC**	1.4 (1.1–1.8)	< 0.0001
**Random effects**		
**Random intercept**	1.4 (0.93–2.0)	
**Residual variance**	0.94 (0.82–1.1)	

Data represent the point estimate (95% confidence interval [CI])

*SNC* standard nasal cannula, *OSNRC* oxygen-sparing reservoir nasal cannula

## Discussion

Here we describe the design and pilot testing of a low-cost OSNRC to augment the delivery of oxygen to pediatric patients with hypoxemia. Pre-clinical optimization of the OSNRC design used a novel, anatomically accurate, artificial respiratory circuit, and demonstrated a FSR of 1.8 to 2.6 under simulated, physiologically relevant conditions. A pilot clinical study demonstrated safety (no observed difference in CO_2_ retention), with no statistically or clinically significant differences in secondary safety endpoints in patients breathing oxygen by OSNRC, compared to SNC alone. Furthermore, clinical efficacy was suggested by the increased SpO_2_ at a given flow rate observed in patients on the OSNRC. The OSNRC achieved a potential increase in SpO_2_ equivalent to an incremental flow rate increase of 0.9 L/min and an FSR of 1.6. Of note, the OSNRC is versatile and could be used with any oxygen supply modality (cylinders or concentrators) that can be used by the SNC.

Several previous studies have examined oxygen-conserving techniques. Generally, these devices were designed for adult COPD patients with chronic hypoxemia in high-income settings rather than children with acute pneumonia in low-resource settings. Transtracheal catheters have a FSR between 2 and 3 in comparison to oxygen administered at the nares [25–27]; however, they have obvious disadvantages associated with an invasive neck surgery and would not be appropriate for pediatric pneumonia in low-resource settings [28]. Commercial DODS systems AccuO2 and CR-50 have FSRs of 9.9 and 2.6, respectively [16, 29], but are prohibitively costly and not yet optimized for pediatrics. Reservoir systems (e.g., Pendant Conserving Nasal Cannula, PNC) [30] had a FSR of 3 in a previous study of adult patients [31]. One study using a lung simulator demonstrated a FSR of 1.1–1.3 for toddler to adolescent simulated patients [32]. In another report on COPD patients, improvement in oxygen saturation with the PNC was 3.3, 4.3 and 3.1% at 0.5 L/min, 1 L/min and 2 L/min oxygen flow rates, respectively [30]. By comparison, our OSNRC improved SpO_2_ by 1.4%, with an FSR of 1.5. Major advantages of reservoir systems include the simplicity and low cost relative to other oxygen-sparing devices. Furthermore, whereas previous devices were designed for adult patients, our OSNRC extends the utility of oxygen sparing reservoir systems to the pediatric age group, who bear a disproportionate burden of global pneumonia mortality.

Patient safety was the primary focus of the pilot study. CO_2_ retention was one possible concern with our apparatus, since the OSNRC re-circulates exhaled air enriched in both O_2_ and CO_2_. Previous studies in COPD patients revealed a small risk of CO_2_ retention during controlled oxygen therapy. However, CO_2_ retention was not observed in our study. Capillary blood gas monitoring demonstrated a correction of hypocapnia as tachypnea resolved with oxygen therapy. Changes in pCO_2_ were no different with the OSNRC than with SNC. Although some cases of clinically insignificant acidosis and hyperlactatemia were observed, these occurred with similar frequency in the OSNRC and SNC groups.

Our study had some limitations. The effectiveness of the OSNRC might have been compromised by improper facial fitting and/or poor tolerability in young children. To accommodate for varying facial profiles, two different sizes of OSNRC were designed, which could be chosen based on patient weight. Despite this, there was one patient for whom the OSNRC was not tolerated during fit testing; however, this was not a major factor in the clinical pilot study. Another limitation of our study was the short duration (1 h) for each period, during which patients were either using OSNRC or SNC, whereas a longer time of observation would be informative. We measured capillary blood gas and transcutaneous pCO_2_; other measurements (arterial blood gases, end tidal CO_2_, FiO_2_) would provide additional information on oxygen treatment and safety with OSNRC. Measurement of alveolar ventilation would also be desirable to directly examine CO_2_ retention. Sample size was limited and the findings of this study should be validated in a larger patient cohort.

## Conclusions

Childhood pneumonia, the leading cause of childhood death globally, as well as other respiratory and cardiac illnesses, may lead to life-threatening hypoxemia. The OSNRC is low-cost and used in a similar manner to commonly used oxygen delivery devices, two important factors for its successful implementation in low- to middle-income countries. Introducing the OSNRC into clinical settings should be accompanied by training for nursing staff on pulse oximetry and accurate titration of oxygen flow rate to maximize the flow savings benefits of the OSNRC. Future directions for the OSNRC include larger clinical studies, using the FSR relative to SNC as a clinical endpoint, as well as possible commercialization of the device. If taken to scale globally, the OSNRC could reduce costs of oxygen supply by reducing oxygen consumption. Efforts to reduce costs and improve efficiency of oxygen delivery could significantly decrease the global burden of acute respiratory disease in children.

## Supplementary information

**Additional file 1: Appendix 1.** Supplementary Methods. **Appendix 2.** Tolerability questionnaire. **Appendix 3.** Supplemental figure.

## Data Availability

The datasets used and/or analyzed during the current study are available from the corresponding author on reasonable request.

## Abbreviations

OCDs: Oxygen conserving devices
OSNRCs: Oxygen-sparing nasal reservoir cannulas
DODS: Demand oxygen delivery systems
COPD: Chronic obstructive pulmonary disease
PNC: Pendant conserving nasal cannula
SNC: Standard nasal cannula
SpO_2_: Peripheral oxygen saturation
3D: Three-dimensional
ETCO_2_: End-tidal CO_2_
FiO_2_: Fraction of inspired oxygen
