# Supplementary material for: Efficacy and safety of oxygen-sparing nasal reservoir cannula for treatment of pediatric hypoxemic pneumonia in Uganda: a pilot randomized clinical trial
Source: BMC Pulm Med. 2020 Aug 31;20:230. doi: 10.1186/s12890-020-01267-8 (PMC7457357; doi:10.1186/s12890-020-01267-8)
Supplement: Supplementary file 1 — Additional file 1: Appendix 1. Supplementary Methods. Appendix 2. Tolerability questionnaire. Appendix 3. Supplemental figure. [file 12890_2020_1267_MOESM1_ESM.doc]

**SUPPLEMENTARY INFORMATION**

**TITLE: Oxygen sparing and clinical safety of nasal reservoir cannula for the treatment of pediatric hypoxemic pneumonia in a low-resource setting**

**Authors’ full names:**

Jerry Mulondo, MBChB, MPH^1^; Stella Maleni, MBChB, MMed^1^, Hellen Aanyu-Tukamuhebwa, MBChB, MMed^2^, Ezekiel Mupere, MBChB, MMed,PhD^2,3^, Alfred Onubia Andama, MBChB, MMed^4^; Chin Hei Ng, PhD^5^; Stephen Burkot, MSc,^5^; Ella M. E. Forgie, BSc^6^; Qaasim Mian^6^; Christine M. Bachman, MPH^5^, Gerard Rummery, BIndDes^7^, Daniel Lieberman, PhD, PE^5^; David Bell, MD, PhD^5,8,†^, Michael T. Hawkes, MD, PhD^6, 9-12,‡,*^, Akos Somoskovi, MD, PhD, DSc^5,*^

**Authors’ affiliation(s):**

^1^Infectious Diseases Research Collaboration, Kampala, Uganda;

^2^Department of Pediatrics and Child Health, Mulago National Referral Hospital and Makerere University, Kampala, Uganda;

^3^Department of Pediatrics, Makerere University College of Health Sciences, Kampala, Uganda;

^4^Department of Medicine, Makerere University College of Health Sciences, Kampala, Uganda;

^5^Intellectual Ventures, Global Good Fund, Bellevue, Washington, USA;

^6^Department of Pediatrics, University of Alberta, Edmonton, Canada;

^7^ResMed Ltd., Bella Vista, Australia;

^8^Independent consultant, Issaquah, WA, USA

^9^Department of Medical Microbiology and Immunology, University of Alberta, Edmonton, Canada;

^10^School of Public Health, University of Alberta, Edmonton, Canada;

^11^Distinguished Researcher, Stollery Science Lab

^12^Member, Women and Children’s Health Research Institute

^†^Current affiliation

^‡^Corresponding author: Michael T. Hawkes, Department of Pediatrics, University of Alberta, 3-588D Edmonton Clinic Health Academy, 11405 87 Ave NW, Edmonton, Alberta, T6G 1C9, Canada, Tel: +1-780-248-5540, Email: [mthawkes@ualberta.ca](mailto:mthawkes@ualberta.ca)

*MTH and AS contributed equally to this paper

**Appendix 1-** *Supplementary Methods*

*Study Setting*

Mulago Hospital is the largest referral hospital in the country, serving both the city population and referrals from other urban and rural hospitals. This 1 500-bed hospital has approximately 761 573 admissions annually, including 9 366 pediatric pneumonia cases. Uganda has the 19^th^ highest under-five mortality rate (U5MR) in the world, with 49 deaths per 1 000 children.^19^ Pneumonia accounts for 11% of the U5MR in Uganda, the fourth highest cause of mortality.^1^

*Pilot study participants*

Children were included in the study if they met the following inclusion criteria: (1) age ≥1 year and ≤6 years of age; (2) severe pneumonia based on World Health Organization (WHO) criteria ^2^; (3) SpO_2_ ≥85% and <94% by pulse oximetry on room air; (4) hospital admission indicated based on clinician judgment; and (5) body weight ≥8kg and ≤26kg. Children were excluded if they had: (1) hypercapnia (pCO_2_ >55mmHg) on room air; (2) pH <7.20 (capillary blood gas); (3) lactate >3mmol/L (capillary blood gas); (4) SICK score ^20^ > 2.4; (5) hemoglobin <7g/dL; (6) facial abnormalities or trauma precluding use of nasal prongs; (7) requirement for intubation or positive-pressure ventilation; (8) suspected or known pneumothorax; (9) hemodynamic instability; (10) failure to respond to oxygen after trial period (SpO_2_ <90%, measured after 10 minutes of oxygen therapy at 2L/min).

*Study outcomes*

The primary focus of the pilot study was clinical safety of the OSNRC. As the primary safety outcome, we examined CO_2_ retention, as measured by the change in pCO_2_ after 1 hour on OSNRC *versus* SNC alone (Period 1). Secondary safety outcomes included: clinical adverse events, capillary blood gas pCO_2_ above normal range (>45mmHg), lactate above normal range (>3mmol/L), acidosis (pH<7.35), and refractory hypoxemia (SpO_2_<90%) despite supplemental O_2_ therapy at any time on OSNRC. In addition, we compared the longitudinal trends in pCO_2_, pH, and lactate to the control group receiving O_2_ by SNC. Secondary efficacy outcomes included oxygen utilization and SpO_2_ at several O_2_ flow rates, compared between OSNRC and SNC. These outcomes were specified *a priori* in the trial protocol. Capillary blood gas was measured using an iSTAT-1 handheld analyzer with CG4+ cartridges (Abbott Point of Care Inc., Princeton, NJ). Pulse oximetry employed the Rad-5® oximeter (Masimo Corp., Irvine, CA).

*Sample size*

We planned to enrol 16 patients to demonstrate safety of the OSNRC according to our primary safety endpoint. To show that OSNRC does not lead to CO_2_ retention, we sought to demonstrate, with 90% power at the α=0.05 level of significance (one-sided), that the change in the pCO_2_ after the first hour of OSNRC therapy was not more than 5mmHg greater than SNC alone (non-inferiority margin). Assuming that the mean (SD) rise in pCO2 is 10 (3) mmHg, standard sample size calculations indicate that 7 patients in each group would be needed to demonstrate non-inferiority.^21-23^ We increased the sample size by 10% to account for possible incomplete data.

*Randomization*

Eligible patients were randomly assigned to one of two groups (Group A or Group B). Randomization by blocks of 4 was employed, in order to balance group assignment over time, using a computer-generated list. Treatment assignment was recorded on paper and kept in sequentially numbered, sealed, opaque envelopes in a locked cabinet. After patient stabilization and informed consent, the next envelope was drawn by a study investigator. The trial was not blinded.

*Study procedures*

At screening, the clinician collected clinical and laboratory data to verify inclusion and exclusion criteria. Screening procedures also included a trial of standard O_2_ therapy and only patients with SpO_2_ above 90% after 10 minutes on 2L/minute O_2_ were eligible for enrolment.

Following baseline assessments, patients were randomized to one of two groups. Groups differed only with respect to the order of the intervention (OSNRC *versus* SNC). Patients in Group A received O_2_ for 1 hour using OSNRC (Period 1), followed by a 1-hr period of continued use of SNC alone (Period 2). The order was reversed for Group B. Patients with a body weight of 8-13 kg were treated with a 30 mL OSNRC, while patients weighing 14-26 kg received treatment with a 50 mL OSNRC. Flow rate of oxygen during Period 1 began at 2L/min and was titrated downward, as allowed, to maintain SpO_2_>94%.

During each treatment period, vital signs were recorded every 15 minutes. At the end of each hour, capillary blood gas (pH, pCO_2_, pO_2_, base excess, HCO_3_ and lactate) was measured. Any adverse events or concomitant medications were also noted by the study investigators.

*Statistical analysis*

Data were collected on paper case report forms and uploaded to a password protected database (KoBo Toolbox, Harvard Humanitarian Initiative, Cambridge, MA). Data quality review was done to ensure that all participants met entry criteria and to generate data queries for correction as necessary. Data analyses were performed using GraphPad Prism version 6 (GraphPad Software, Inc., La Jolla, CA, 2012), and *R* (R Core Team, Vienna, Austria). Descriptive statistics for dichotomous data used number and percentage, and continuous data were summarized as median and interquartile range (IQR) or mean and standard deviation (SD), as appropriate.

For the primary safety endpoint, we computed the difference in means of the change in pCO_2_ over Period 1 between OSNRC and SNC, together with 95% confidence interval (CI) and concluded non-inferiority if the upper limit of the 95% CI was less than a margin of +5%.

For secondary endpoints, we compared patients receiving O_2_ by OSNRC and SNC in Period 1 and Period 2 of the cross-over study separately. Comparative statistics used the chi-squared test or Fisher exact test for count data, as appropriate, and Mann-Whitney U test (non-parametric) for continuous variables. For one efficacy endpoint, we examined differences in SpO_2_ between OSNRC and SNC, adjusting for oxygen flow rate and study period. We used a linear mixed effects (LMEs) model to account for repeated measurements of SpO_2_ over time using *R* and *lme4.* As fixed effects, we entered device (OSNRC *versus* SNC), oxygen flow rate, and study period without interaction term into the model. We modeled SpO_2_ as a linear function of flow rate, with random intercepts and slopes for each subject. Visual inspection of residual plots did not reveal any obvious deviations from homoscedasticity or normality. P-values were obtained by likelihood ratio tests of the full model against the model without device (OSNRC *versus* SNC).

**Appendix 2**-*Tolerability questionnaire*

**MASK FIT TEST
HEALTH CARE WORKER QUESTIONNAIRE**

**STUDY TITLE:** A Feasibility Study Evaluating a Novel Mask (Nasal Reservoir Cannula) Plus Standard Nasal Cannula vs Standard Nasal Cannula Alone for Supplemental Oxygen Delivery in the Treatment of Hospitalized Pediatric Patients with Hypoxemia Due to Severe Pneumonia

**DATE: __/__/__
PATIENT DATA:**Patient ID number: **____________**

Date of Birth: __/__/__ Weight: __________kg
D/ M/ Y Gender: **_** **Male _ Female**

Body weight 8-13 kg _ **Yes ---------------------------------**🡪**Small mask**

Body weight 14-26 kg _ **Yes ----------------------------------**🡪**Large mask**

Instructions to health care worker:

Health care worker is to place the mask and face band on the child and have them remain with the mask on for up to 5 minutes.

Does the mask fit the patient’s nose appropriately? **_ Yes _ No**Can the patient tolerate the mask? **_ Yes _ No**Is the placement of the mask onto the patient easy to perform? **_ Yes _ No**

Can the patient tolerate the face band? **_ Yes _ No**Is the placement of the face band onto the patient easy to perform? **_ Yes _ No**

Is the size of the mask appropriate? **_ Yes _ No**

Once all questions have been answered, the health worker can proceed to check other patients.

**Appendix 3**-*Supplemental figure*

**Figure S1. A and B.** Capillary blood gas pH changes were similar in OSNRC and SNC groups. With resolution of tachypnea and respiratory alkalosis, pH decreased in both groups, consistent with an unmasked underlying metabolic acidosis. **C and D.** Changes in lactate levels were similar in both groups. Elevated lactate was observed in a small number of patients in both groups and may be related to sample processing since other blood gas parameters were unremarkable. **E and F**. Base excess was unchanged over the short (1 hour) trial periods, indicating an underlying compensatory or concomitant metabolic acidosis.
